# Unnatural Deaths among Autopsy Cases Brought at Tertiary Care Hospital of Western Nepal during COVID-19 Pandemic Period: A Descriptive Cross-sectional Study

**DOI:** 10.31729/jnma.6484

**Published:** 2021-12-31

**Authors:** Santosh Koirala, Krishna Subedi, Nuwadatta Subedi

**Affiliations:** 1 Department of Forensic Medicine, Chitwan Medical College, Chitwan, Nepal; 2 Department of Community Dentistry, Gandaki Medical College Teaching Hospital and Research Centre, Pokhara, Nepal; 3 Department of Forensic Medicine, Gandaki Medical College Teaching Hospital and Research Centre, Pokhara, Nepal

**Keywords:** *autopsy*, *COVID-19 pandemic*, *deaths*, *suicide*

## Abstract

**Introduction:**

The outbreak of COVID-19 has changed patterns of mortality in different setups. The rate of suicide has increased in some countries during the pandemic while the overall death rates have decreased. The study was conducted with objective to find out the prevalence of unnatural deaths among the autopsy cases brought at tertiary care hospital during COVID-19 pandemic period.

**Methods:**

This is a descriptive cross-sectional study using the records of the medico legal autopsies conducted from 24th March 2020 to 23rd August 2020 during the COVID-19 pandemic in Pokhara Academy of Health Sciences. Ethical approval was taken from Institutional Review Committee of Pokhara Academy of Health Sciences (Reference number 28.2077/78). Whole sampling method was used. Records which were available were included in the study whereas those cases whose complete records were not available were excluded. Point estimate at 95% confidence interval was calculated along with frequency and proportion for binary data.

**Results:**

Out of 188 deaths studied at the autopsy during the COVID-19 pandemic period, the prevalence of unnatural deaths was 147 (78.19%) (71.04-85.33 at 95% Confidence Interval). Among these deaths, 109 (74.14%) were males and 38 (25.85%) were females. Suicide was the most common manner attributing to 78 (53.06%) of the unnatural deaths.

**Conclusions:**

The prevalence of suicide was more than those demonstrated by earlier observations in similar settings before the pandemic period. Suicidal deaths were more common during the COVID 19 pandemic. This is an indicator of frustration of the people and necessary steps have to be taken to decrease such deaths in similar conditions to come.

## INTRODUCTION

The COVID-19 pandemic has caused to an unparalleled increased in the death rates throughout the globe and the United Kingdom, the United States, Italy, India are mainly affected.^1^ The outbreak of COVID-19 has changed patterns of mortality in different set ups and the death rates have been drastically increased during the pandemic.^2,3^ The rate of suicide has increased in some countries during the pandemic^4^ while the overall death rates decreased though the rate of homicides among males increased in other settings.^5^

In the context of decreased productivity and psychological stress, many countries have been supporting their citizens with health care and various relief funds while some developing countries, including Nepal lack this aspect.^6,7^

The study was conducted with objective to find out the prevalence of unnatural deaths brought for autopsy at Pokhara academy of health sciences during COVID-19 pandemic period.

## METHODS

This is a descriptive cross-sectional study conducted at Pokhara Academy of Health Sciences, Western Regional Hospital, Pokhara, a tertiary level government hospital of Gandaki Province, Nepal. The hospital is a major centre to perform medico legal autopsies in the province and it serves as a referral centre from the hospitals of adjoining districts too. The duration of study was from October to December 2020. Ethical clearance was taken from Institutional Review Committee of Pokhara Academy of Health Sciences (Reference no 28.2077/78).

The sample size was calculated by using the formula

n = Z^2^ × p × q / e^2^

  = (1.96)^2^ × 0.5 × (1-0.5) / (0.08)^2^

  = 151

Where,

n= required sample size
Z= 1.96 taken at 95 % Confidence Interval (CI)
p= prevalence taken as 50% for maximum sample size.
q= 1-p
e= margin of error, 8%

Adding 10% for non-response rate, we get a sample size of 162. Whole sampling was done and sample size of 188 was taken.

We reviewed the records of the medico legal autopsies conducted from 24th March 2020 to 23rd August 2020 during the COVID-19 pandemic period. We included of all the cases available in the study duration and whose records were available whereas those cases whose complete records were not available and unidentified bodies were excluded.

The data was collected in a structured proforma designed for the study. The requisition letter to perform a medico legal autopsy, the inquest papers and the autopsy report were the source of the data. The cause of death was identified from the autopsy reports and the manner of death was based on the inquest and the autopsy reports. Statistical Package for Social Sciences version 16 and Microsoft Excel were used for data entry and analysis. Point estimate at 95% confidence interval was calculated along with frequency and proportion for binary data.

## RESULTS

Out of 188 deaths studied at the autopsy during the COVID-19 pandemic period, the prevalence of unnatural deaths was 147 (78.19%) (71.04-85.33 at 95% Confidence Interval). Among these deaths, 109 (74.14%) were males and 38 (25.85%) were females (Table 1).

**Table 1:** Deaths during COVID-19 pandemic period (n=188).

Cause of death	n (%)	Males n (%)	Females n (%)
Unnatural Deaths	147 (78.19)	109 (74.14)	38 (25.85)
Sudden Natural Deaths	31 (16.5)	22 (70.96)	9 (29.03)
Undetermined	10 (5.3)	6 (0.6)	4 (0.4)
Total	188 (100)	137 (72.87)	51 (27.12)

The causes of death in the study duration and their distribution with respect to gender is represented below (Table 2). It was evident that the most common cause of death was hanging in 65 (44.21%) cases. It was followed by accidental deaths (other than road traffic accidents and drowning) in 41 (27.89%) cases.

**Table 2:** Causes of death among unnatural deaths during COVID-19 pandemic period (n=147).

Cause of death	Total n (%)	Females n (%)	Males n (%)
Hanging	65 (44.21)	24 (36.92)	41 (63.07)
Road Traffic Injuries	16 (10.88)	3 (18.75)	13 (81.25)
Other Accidental	41 (27.89)	6 (14.63)	35 (85.36)
Drowning	17 (11.56)	2 (11.76)	15 (88.23)
Poisoning	8 (5.44)	3 (37.5)	5 (62.5)
**Total**	147 (100)	38 (25.85)	109 (74.14)

The prevalence of suicidal deaths and accidental deaths was found to be 78 (53.06%) and 65 (44.21%) respectively. Homicidal deaths were seen in 3 (1.36%) (Table 3).

**Table 3:** Manner of death during COVID-19 period (n = among unnatural deaths 147).

Manner of death	n (%)
Accident	65 (44.21)
Suicide	78 (53.06)
Homicide	2 (1.36)
Undetermined	2 (1.36)
**Total**	147 (100)

## DISCUSSION

The government of Nepal enforced a strict lockdown from 24 March 2020 which was eased partially on 11 June 2020. The end of lockdown with just few restrictions was on 22 July 2020.^8^ Again lockdown was enforced on 19 August 2020^8^ which was extended up to 2nd September 2020 with few restrictions.^9^ When the COVID-19 pandemic was spreading, many countries opted lockdown as a measure to control the spread of pandemic. This was proven to be a useful strategy to limit the spread.^10^ The lockdown decreased the mobility of the people and it also increased financial burdens to the peoples.^10^

In Nepal, the first case of COVID-19 was detected on Jan 13, 2020, in a 32-year-old Nepalese student from Wuhan, China.^11^ Since then, the cases kept on increasing and by April 29st 2021, the number of cases reached to 312,699 and 3,211 deaths.^12^ The strict lockdown was criticised by many people and condemned to be one of the factors causing deaths due to other reasons than COVID 19.^13^ The lockdown has been alleged to have caused improper delivery of maternity services which has subsequently increased maternal deaths. It is also regarded as one of the culprits of poor mental health and increase in suicidal deaths.^7^

Our study showed more prevalence of suicides in COVID-19 pandemic period than other manners. The prevenance of suicide in autopsy series was less than that accidental deaths during the pre-COVID time in an observation from Dharan.^14^ The rate of suicide was 36.2% in a study from an autopsy series in a tertiary hospital of central Nepal,^15^ which was lower than that in our finding. Similarly, suicides were attributed to 25% of total autopsies from a major autopsy center of the capital city of Nepal.^16^ Deaths due to suicides are based on various problems such as stress, job loss, domestic violence, torture, prolonged disease conditions and other psychiatric illness. Our study shows that the prevalence of suicide was piled up in the COVID-19 pandemic period, the secondary causes of which may be attributed to stress, fear of symptoms causing from COVID-19, the chaos of fear of death if infected by the virus, poor financial conditions and difficulty in access to health care facilities due to strict lock down period as imposed by the government.

The exact reasons for committing suicides during COVID-19 period needs a careful monitoring to prevent it in future other unexpected pandemics so that we can be aware as death of a single person in low socioeconomic status cost much to the family. The increased suicides in lock down due to COVID-19 in older adults has been attributed to the feeling of adverse effects of isolation.^17^

Death, considered as a social phenomenon, has been disrupted for those who are dying in hospital and for those who are dying at home. In the remaining days of the year 2021 AD, our ability to react to a pandemic will also be tested by how we are able to maintain the social dimension of death and dying. Although our assumptions for this were speculative, we are confident that, if pandemic era prolongs, death due to suicide shall contribute greatly to mortality. The drive of stress and poverty might lie outside of the health system, but there are interventions that health policy makers and the government could consider easy access to health care emergency needs, free food and low tax imposition. More importantly, multisectoral action should be taken to mitigate increases in suicides by strengthening and expanding social safety nets, supporting agricultural and financial systems etc.^18^

The study has explored the pattern of deaths during the COVID 19 pandemic period which included the period of lockdown which is the strength of the study. The limitations include the non-coverage of overall pattern of deaths in the broader population to generalise to the national status.

## CONCLUSIONS

The prevalence of suicide was more than those demonstrated by earlier observations in similar settings before the pandemic period. The study has demonstrated suicide as the most common manner of deaths during the COVID-19 pandemic. This is an indicator of stressful conditions of the people and necessary steps have to be taken to decrease such deaths in similar conditions to come.
